# Shaping the future-ready doctor: a first-aid kit to address a gap in medical education

**DOI:** 10.5116/ijme.5fad.2d3a

**Published:** 2020-11-30

**Authors:** Farah Otaki, Nerissa Naidoo, Saba Al Heialy, Anne-Marie John-Baptiste, Dave Davis, Abiola Senok

**Affiliations:** 1 Strategy and Institutional Excellence, Mohammed Bin Rashid University of Medicine and Health Sciences, Dubai, United Arab Emirates; 2 College of Medicine, Mohammed Bin Rashid University of Medicine and Health Sciences, Dubai, United Arab Emirates; 3 Center for Outcomes in Medical Education, Mohammed Bin Rashid University of Medicine and Health Sciences, Dubai, United Arab Emirates

**Keywords:** Future-ready doctor, gap in medical education, United Arab Emirates

## To the Editor

With the advent of Industry 4.0 (i.e., the fourth industrial revolution) came a paradigm shift built on core work-related skills that further dictated the emergence of a "new" world, with diverse professional disruptors and innovators at the forefront.^1^ In an effort to address this new paradigm, recommendations in higher education for the "creation of more practical and applied curricula" and for enhancing "relationships between higher education institutions, employers, and other partners . . ." have been proposed.^2^ To create a level playing field for global healthcare sectors of the future, Morrison^3^ outlined the diverse roles a future-ready doctor will be expected to uphold. Thus, medical education has to evolve to mold a holistic future-ready doctor who can treat and continuously innovate.^4^ To this end, medical schools are challenged to amalgamate basic medical sciences with clinical sciences seamlessly and to adapt their curricula to yield millennial physicians who are able to respond to and act on current and emerging trends in healthcare.^5^ In addition to building on Flexner's legacy^6^ to ensure progressive pedagogical approaches, innovative means to incorporate the active components of human presence, comparable to that of core work-related attributes (i.e., heart - values, head - knowledge, mind - qualities, and hands - skills), need to be developed.^7^ However, it is undeniable that traditional classroom instruction alone cannot produce these characteristics of the future-ready doctor.

Co-curricular programs, which are out-of-classroom experiences, offered either independently from or in conjunction with the academic curriculum, hold a lot of potential for developing these key work-related attributes among students. Such programs can be based upon Kolb's Experiential Learning Theory (ELT), which is a pedagogical model, that entails a four-stage learning cycle of concrete experience, reflective observation, abstract conceptualization, and active experimentation.^8^^,^^9^ To further maximize the impact of these programs, it is worth anchoring them in holistic theories of education (e.g., situated learning), which also take into consideration the individual's participation and context of learning.^10^

On the basis of a deep-rooted institutional philosophy that "knowledge has no limits and hence learning should have no boundaries", the Mohammed Bin Rashid University of Medicine and Health Sciences (MBRU), Dubai, United Arab Emirates, developed and has been implementing a co-curricular program for medical students, based on Kolb's ELT and situated learning theory, called the MBRU-Summer Scholars Program (SSP). Since its inception in October 2017, the MBRU-SSP has been upholding the institutional vision and mission, and values' system, as well as complementing the spiral curriculum in the medical school. The key objective of the program is to provide a platform for students to acquire and integrate skills, knowledge, and competencies in research, clinical practice, community service, health systems, and/ or arts and culture domains. The program is characteristically diverse, giving participants the autonomy to select from an extensive array of offerings, thus setting MBRU-SSP apart from others which tend to focus on single domains.^11^^-^^13^ In addition, it provides cross-cultural out-of-classroom experiences to increase the students' capacity for creativity and critical and lateral thinking, and enhance their interpersonal skills. The MBRU-SSP annual cycle entails three phases: preparation, implementation, and evaluation. In addition, there is a post-placement awards' day, during which students reflect upon their experiences by showcasing their work through poster presentations. Throughout the program cycle, there exists substantial engagement of all involved stakeholders. This expands on the strengths and resources within the MBRU community and facilitates collaborative partnerships throughout all phases of the program. To date, the MBRU-SSP has placed students in 40 national and international collaborating centers across 11 countries. The duration of students' placements ranges from 1 to 6 weeks and occurs during the summer months.

As part of the evaluation phase of the 2018/2019 MBRU-SSP cycle, quantitative and qualitative data was collected from three groups of stakeholders: participating students, onsite mentors, and program organizers. In general, all stakeholders appreciated the MBRU-SSP. Majority of the participating students rated the overall quality of the experience as excellent. The students found the program to be valuable to them as individuals (personally and professionally, and academically), and to the community-at-large. Personal development, entailing character and resilience building, emerged as a significant benefit. Opportunities to apply previously acquired academic knowledge and expand students' professional network were highlighted. Students also conveyed that the impact of the MBRU-SSP extends to the community through the program's contribution to institutional advancement of MBRU and hosting centres. From the perspective of the MBRU-SSP organizers, the program objectives were achieved and contributed to the nurturance of global citizens. Onsite mentors expressed satisfaction with the overall organization of the program, as well as the attendance and level of engagement of the participating students.

The MBRU-SSP represents a co-curricular program, grounded on holistic educational theories, which may serve as a so-called "first-aid kit" to humanize medical education, building on Flexner's legacy, and nurturing a future-ready doctor. The program fosters learning that occurs via individual adaption, along with participation in the real world and interaction with the wider society. It offers an example of a reproducible co-curricular program with positive impact on participants as individuals within the context of their interconnected communities.

### Conflict of Interest

The authors declare that they have no conflict of interest.
